# Negative emotion can be “more negative” for those with high metacognitive abilities when problem-solving

**DOI:** 10.3389/fpsyg.2023.1110211

**Published:** 2023-03-13

**Authors:** Seok-sung Hong, Jinhee Bae, Lisa K. Son, Kyungil Kim

**Affiliations:** ^1^Social Science Institute, Ajou University, Suwon, Republic of Korea; ^2^Brain Science, Institute Korea Institute of Science and Technology (KIST), Seoul, Republic of Korea; ^3^Department of Psychology, Barnard College, Columbia University, New York, NY, United States; ^4^Department of Psychology, Ajou University, Suwon, Republic of Korea

**Keywords:** metacognition, monitoring ability, emotion, cognitive reflection test, problem-solving strategies

## Abstract

**Introduction:**

Metacognitive monitoring ability enables you to learn and solve problems more efficiently through appropriate strategies. At the same time, those who are high in monitoring ability are known to allocate more cognitive resources to the perception and control of negative emotions, as compared to those with low metacognitive ability. Therefore, while monitoring emotions may help reduce the negative emotion by enabling efficient control, it could also interrupt the use of an efficient strategy when problem-solving, as cognitive resources may be depleted.

**Methods:**

To confirm this, we divided participants into groups with high and low monitoring abilities and manipulated emotions by presenting emotional videos. Subsequent to the manipulation, problem solving strategies were examined using items from the Cognitive Reflection Test (CRT).

**Results:**

Results showed that those who were high in monitoring ability were shown to use more efficient problem-solving strategies than those who were lower in monitoring ability, but only in situations when positive or no emotions were manipulated. However, as hypothesized, when negative emotion was aroused, the CRT scores of high monitoring ability group were significantly lowered, decreasing to the same performance as those with low monitoring ability. We also found that metacognitive monitoring ability, when interacting with emotion, indirectly affected CRT scores, and that monitoring and control, when affected by emotion, were mediated in the process.

**Discussion:**

These findings suggest a novel and complicated interaction between emotion and metacognition and warrant further research.

## 1. Introduction

When faced with a problem, it is in our nature to solve it. To solve any problem, however, we first need to understand what the problem is. When we feel a stomach ache, we ask ourselves, “was it something I ate?” When our goal is to win, we weigh our chances of winning. In an educational context, the process is similar. When we face an exam question, but cannot immediately come up with the answer, we take a step back, perhaps reread the problem, to gain a clear assessment of what is being asked. Only when a clear assessment is reached are we to move towards a potential solution, by employing appropriate strategies (Reed and Bolstad, 1991). The process of making an assessment of the problem, by accumulating sufficient information, is the first step in *metacognition*, also known as *monitoring* (Yeung and Summerfield, 2012). One’s monitoring is said to be high when they are able to judge the contents of their cognition accurately. The current research investigated the stability of an individual’s monitoring process by measuring monitoring accuracy across different emotional states.

### 1.1. Monitoring

Metacognition has been said to require two distinct but related processes: *Monitoring* and *control* (Nelson and Narens, 1994). The former refers to the ability of an individual to check their cognitive status and detect errors (Jameson et al., 1990). In a layperson’s description, monitoring is the notion that we can look into our own minds and know what we know and what we do not know. This knowledge then allows us to control our subsequent behavior. For instance, if we know that we do not yet know some to-be-learned information (monitoring), we are able to determine that more study is required (control). If the monitoring process breaks down—for instance, we do not know that we do not know—then we have little chance of seeking the necessary knowledge to fill the gap (Nelson and Narens, 1994).

Not surprisingly, monitoring accuracy, or *sensitivity,* is essential for decision-making and diverse problem-solving (Galvin et al., 2003; Evans and Azzopardi, 2007; Fleming et al., 2010; Maniscalco and Lau, 2012). Research on individual differences found that the more accurate one’s monitoring ability, the more effective one’s problem-solving strategy (Delclos and Harrington, 1991; Davidson and Sternberg, 1998; Halpern, 1998). In addition, given its link to the control process, accurate monitoring also led to higher test performance (Nelson and Dunlosky, 1991; Dunning et al., 2003; Metcalfe and Kornell, 2003; Thiede et al., 2003; Metcalfe, 2009). For example, in Thiede et al. (2003), participants were asked to read, rate their comprehension for, and then answer test questions for a series of texts. In one experimental condition, participants were instructed to write a list of keywords for each text. Results showed that those in the keyword condition had higher monitoring accuracy and performance than those in the non-keyword control condition. Presumably, the higher one’s sensitivity, the better one is at regulating their subsequent behavior, resulting in boosted performance (Thiede, 1999).

The monitoring process, therefore, has been found to be crucial when solving problems (Davidson and Sternberg, 1998; Galvin et al., 2003; Evans and Azzopardi, 2007; Fleming et al., 2010; Maniscalco and Lau, 2012). In Davidson and Sternberg (1998), the researchers asked participants to answer the following problem—Tom’s mother had three children. She named the first one Penny and the second one Nickel. What did she name her third child? A good number of participants answered “Dime,” likely because Penny reminded them of “money” and Nickel of “nickel-and-dime.” The authors posited that, in general, metacognitive abilities first allowed participants to appropriately encode the components of a problem and to form a mental model or representation of its elements. They also stated that these abilities were an important skill involved in selecting appropriate plans and strategies and identifying or eliminating obstacles to problem-solving—all comprising the full metacognitive monitoring and control process. Again, an accurate monitoring process is likely to give rise to more efficient strategies, resulting in more successful solutions. Indeed, when one knows that one does not know, one can select that strategies that would help fill that gap, such as allocating more study time, and, as a result, increase later test performance (Metcalfe and Finn, 2008; Pourmohamadreza et al., 2011).

While monitoring accuracy is crucial, there may be factors that “shift” one’s assessment of their own knowledge. Some studies have shown, for instance, that simply by presenting a prime, one’s judgments of what they know can be made overconfident (Schwartz and Metcalfe, 1994). And on the flipside, other studies have shown that feedback might decrease people’s monitoring judgments on a subsequent task (Koriat, 1997; Finn, 2008). In these studies people are at first overconfident, but then after taking a test, and presumably realizing that they did not know as much as they thought they had known, their following judgments shift to being more underconfident. That is, people’s *memory-for-past-test* allows them to know that they might not know. In the current research, we sought to examine if positive or negative emotional states would also shift one’s monitoring accuracy.

The shift in monitoring has been a question on interest for decades. In large part, when the science of metacognition took off, the accuracy of one’s monitoring system was the primary question. This seemed to be so because, unfortunately, the monitoring system was consistently inaccurate. Numerous studies presented data showing that we suffered from overconfidence, consistently judging our learning to be higher than could be actually proven on tests, as well as underconfidence when it came to our faith in learning information from particular subfields such as math or science—resulting in a common new phrase “math anxiety.” No doubt such shifts in monitoring would also affect control decisions. Indeed, it is not always possible to pull them apart. For example, if someone decided to drop out of a course—would it be because they were not allocating sufficient time to their studies? Or would it be because they felt underconfident about their potential? Or could it be both? Regardless, one’s monitoring assessments need to be accurate if learners are to have any chance of making good decisions. In Bjork’s 1994 review of the literature to that point on monitoring, a meta-analysis showed that the average correlation between our judgments of knowledge and our actual knowledge, was barely positive, falling at meager 0.2. Given the shakiness of our monitoring accuracy, it continues to be critical to uncover the mechanisms behind the shift, and the current approach is to examine its relation to emotional states.

### 1.2. Effects of emotion

Considerable research has shown that emotional states are likely to influence problem-solving (Teigen, 1994; Spering et al., 2005; Trezise and Reeve, 2014). While evidence has shown that some amount of stress might enhance performance, on the whole, negative emotions seem to impede successful problem solving. For example, when negative emotions are aroused, say, through heightened stress or anxiety, cognitive resources including working-memory seem to be continuously used to identify and to regulate those negative emotions—aligning with the math anxiety issues mentioned above. In effect, insufficient cognitive resources necessary for task performance remain (Eysenck and Calvo, 1992; Beilock et al., 2004; Baddeley, 2012; Plass and Kalyuga, 2019). In other words, people seem to monitor the emotions that they are feeling, leading to poor encoding of the problem at hand (Isen et al., 1987; Crooks and Alibali, 2013), and, consequently, bad performance. Some hopeful research, however, has shown that employing various control strategies, including a change in thinking or goals, or providing an explanation for why the emotional state was induced, have been found to reduce negative emotions (Harris et al., 1981; McCoy and Masters, 1985; Davis et al., 2010). Moreover, when a positive emotion is aroused, data have shown there to be a positive effect on performance during decision-making, negotiation, and problem-solving tasks (Isen et al., 1985; Hirt et al., 1996). Whether these emotional states affect monitoring and/or control during metacognitive processing is the question we address here.

The classic Yerkes-Dodson Law encapsulates the conundrum presented above. There seems to be a case for the notion that some amount of stress, or, say, curiosity, would be beneficial for learning and for problem solving (Faller et al., 2019; Khazaei et al., 2021). On the other hand, too much stress or negative emotions could disrupt the process by stealing resources away (Gustems-Carnicer et al., 2019; Plass and Kalyuga, 2019; Zhang et al., 2019). More recently, Ko et al. (2020) gave participants working memory, concentration, short-term memory, planning, and divergent thinking tests. The participants performed the tasks in two offices representing two emotion conditions: offices with windows (positive emotion condition) and offices without windows (negative emotion condition). Results showed that, for a few tasks, people performed better when working in the “positive” offices than in the “negative” offices.

In addition, Gottman et al. (1996) suggested that metaemotion, “emotion about emotion,” and metacognition, “cognition about cognition,” are parallel in that they are involved in the executive control of emotion and cognition, respectively. Based on their study, they concluded that the two cognitive activities could conflict (Norman and Furnes, 2016), and other studies eventually confirmed this (Legrand et al., 2020; Pennequin et al., 2020). According to the classic “Schachter-Singer theory” (Schachter and Singer, 1962), monitoring one’s cardiac signals (e.g., change in heart rate) induced an emotional state. That could mean that when people experience an emotional stimulus, monitoring of one’s cardiac signals, such as the change in heart rate, may occur. In other words, resources may be more quickly depleted when in an emotional state than when in a neutral state. Indeed, monitoring of the task (in this case, a word recognition metamemory task) led to less accurate performance when participants were asked to monitor negative emotions than positive ones (Legrand et al., 2020).

When comparing people with relatively high monitoring ability and low monitoring ability, a number of studies have shown that the former group of people perceived negative emotions more strongly (Hudlicka, 2005; Spada et al., 2008). Essentially, individuals with high monitoring ability were thought to have a high level of emotional perception because they aimed to “solve” their emotional states and achieve inner peace, that is, to escape or to avoid that negative emotion. In addition, Spada et al. (2008) reported that those with higher monitoring abilities showed higher levels of stress perception and higher levels of negative emotion. Taken together with the above data, how emotion relates to monitoring seem complex—in some cases, negative emotions may interfere with good monitoring; in others, good monitoring can mean an eventual decrease in negative emotion. Whether this discrepancy can be rectified, perhaps *via* increased awareness of metacognitive feelings of confidence (FOC), was the aim of the current study.

The accumulating data seem to suggest, in the least, that improving monitoring accuracy may help the individual in escaping negative emotional states by helping them to select appropriate control strategies. On the other hand, having those individuals allocate additional resources for improving monitoring may backfire, interfering with finding information or strategies needed to solve the task (Clore and Huntsinger, 2007, 2009; Pekrun and Stephens, 2009; Tsai and Young, 2010; Pennequin et al., 2020). In other words, when participants experience negative emotions, those with high metacognitive ability might tend to focus more on their emotions and respond sensitively, which, in turn, may temporarily *lower* the accuracy of their monitoring ability (and as a result, lead them to select sub-optimal problem-solving strategies). For example, imagine a warning system that sounds when a window shakes. If the sensitivity of the sensor is appropriate, the alarm will give warning when someone shakes the window by force. However, if the sensor is overly sensitive, the warning sound will go off even with a light wind breeze. This means that unlimited sensitivity is not always good, and can even look like a malfunction. Likewise, we hypothesized that an individual with good monitoring ability will not always mean the one with the highest sensitivity. Of course, high monitoring ability is likely to have high control ability, so negative emotion is likely to be recovered if given some time (Nguyen et al., 2018). Still, our goal was to find out if there was such a limit on “in the moment” sensitivity when perceiving emotions when it came to monitoring abilities. The reason for doing so was in following with the data coming out of the literature looking at online brain activity.

Studies of monitoring in tandem with brain activity have been continuously increasing. Over the past decade or so, research has shown that people with high-monitoring sensitivity tend to allocate more cognitive resources for correct judgments (Fernandez-Duque et al., 2000; Shimamura, 2000; Overbeek et al., 2005; Fleming et al., 2010; Simons et al., 2010; Fleming et al., 2012; Kepecs and Mainen, 2012; Yeung and Summerfield, 2012). Metacognitive ability has been known to be closely related to functioning in the medial prefrontal cortex (PFC), and as monitoring sensitivity increases, activation of the prefrontal lobes increases as well (Kepecs and Mainen, 2012; Yeung and Summerfield, 2012). It was also observed that the lateral prefrontal cortex (Fleming et al., 2010) and anterior cingulate cortex (Hester et al., 2008, 2009) play important roles in monitoring, suggesting that simultaneous activation occurs in various areas of the brain (Overbeek et al., 2005; Lau and Passingham, 2006; Yeung and Summerfield, 2012). Also, experiencing negative emotions activates the medial PFC and ACC (Etkin et al., 2011). On the whole, the data suggest that metacognition and negative emotions share similar brain activation areas, and that if both cognitive activities occur at the same time, people may become overloaded.

In summary, the ambiguous relationship between monitoring and emotion is what we address here. On the one hand, a person with a high level of monitoring sensitivity may seem to use more resources, presumably to process emotions, as studies have shown. On the other hand, other studies suggest a positive relation between metacognition and emotional perception (Hudlicka, 2005; Spada et al., 2008; Tajrishi et al., 2011). We set out to resolve this ambiguity, and to investigate how monitoring abilities would guide problem-solving, when positive and negative emotional states came into play. We hypothesized that given a possible limitation of resources that would affect sensitivity, even those high on monitoring ability would be less likely to succeed on a problem-solving task when put in a negative emotion state.

## 2. The current study

In this study, we explored the relationship between one’s emotional state and performance on a cognitive task. Specifically, we were interested in rectifying the conflicting results in the literature, by exploring the impacts on one’s metacognitive monitoring ability. While those with relatively skilled monitoring ability may be likely to use more optimal problem-solving strategies, we also may assume that they are using more cognitive resources—to monitor their cognitions and emotions. Then, during an emotional task, it might be that those high in monitoring ability, and *sensitivity*, might show reduced performance, if, indeed, their resources were being depleted by monitoring their emotions. This may seem especially so for negative emotional states as compared to positive emotional states. It would be interesting to see if those high in monitoring ability would tend to monitor their emotions more than those low in monitoring ability, resulting in, ironically, disrupted problem-solving success. While we remained open in our expectations, we hypothesized what we wondered above—that the effects of emotion, particularly negative emotions, would be further complicated by one’s (high) monitoring abilities. Thus, while negative emotions will lead to lower performance on problem-solving tasks, these disruptions might be amplified in those higher in monitoring sensitivity.

In Experiment 1, a cognitive reflection test (CRT) was used to check whether deliberate problem-solving strategies varied according to the level of metacognitive monitoring ability (high versus low).

The hypothesis was as follows:

*H1*: People with high metacognitive monitoring ability would have a higher correct answer rate than those with low level metacognitive monitoring ability.

In Experiment 2, to test the potential effects of emotional state on performance, and to see if there is a difference between groups according to level of metacognitive monitoring ability, prior to solving the CRT, we manipulated positive and negative emotions by showing video clips. As a result, the following hypotheses were made:

*H2*: People with high metacognitive monitoring ability would have a higher correct answer rate than those with low level metacognitive monitoring ability in the positive emotion condition.

*H3*: People with high metacognitive monitoring ability would have a lower correct answer rate than or be similar to those with a low-level metacognitive monitoring ability in the negative emotion condition.

*H4*: Correct answer rate would be affected by negative emotions differently across monitoring ability, which, in turn, would affect control decisions, and solving success.

The reason for using the cognitive reflection test was because the problems appear, at first glance, to be fairly easy, allowing individuals to believe that they can be quickly solved (an intuitive strategy). However, the problems are designed to be solvable only by using a more deliberate strategy (Frederick, 2005)—where cognitive resources are necessary. Moreover, CRT, which requires individuals to choose a particular appropriate metacognitive process in order to problem-solve, can be used as measure of monitoring ability. High success on the CRT has been found to be correlated with metacognitive abilities (Toplak et al., 2011). Since there has been no direct research on whether CRT can be used to evaluate metacognitive ability, this was our goal in Experiment 1.

## 3. Experiment 1

### 3.1. Method

#### 3.1.1. Participants

One hundred undergraduate students $(M_{age}=22.52$, $N_{male}=45$, $N_{female}=55)$ attending Ajou University (South Korea) participated in this experiment. All participants signed consent forms prior to starting the experiment, and received course credit or gift cards for their participation.

#### 3.1.2. Materials

##### 3.1.2.1. Word-pair learning task

The Word-pair learning task was used to measure general metacognitive ability. Pairs were formed by selecting from a list of 60 concrete Korean nouns. To vary the difficulty, one-third of the word-pairs consisted of items from the same category—highly associated, *easy* pairs (e.g., cat—mouse); another one-third were of the same category, but were not associated—intermediate pairs (e.g., banana—plum); the remaining third were items selected from different, unrelated categories—difficult pairs (e.g., boat—pen). In a pilot test, the Difficulty level was checked by measuring both the ease of learning (EOL) judgments, and the accuracy of memory on a cued-recall test. Twenty pairs taken from each of the difficulty levels were presented one at a time, for 5 s each. Then, participants were asked to make an ease of Learning (EOL) judgment by indicating how difficult each pair would be to learn. Participants responded by selecting one of six confidence ratings ranging from most difficult to learn to easiest to learn of (0, 20, 40, 60, 80, and 100%). After studying all of the pairs and rating their EOLs, participants were given a free cued-recall test where the task was to type the associated word in the blank, after seeing the single word on the left (e.g., chair—____). The EOL and accuracy results are shown in Table 1.

**Table 1:** EOL and accuracy of paired-associates (pilot test).

	Easy	Mid	Difficult
EOL (%)	78.00	36.60	27.48
ACC (%)	76.54	27.60	14.90

##### 3.1.2.2. Cognitive reflection test

Seven CRT questions, three taken from Frederick (2005), and four taken from Toplak et al. (2014) were selected for the experiment. These questions were used to measure accuracy in problem-solving. All questions were translated into Korean for presentation.

#### 3.1.3. Procedure

The overall procedure is presented in Supplementary Figure 1. Once individuals arrived, the experimenter stated to participants that they would be taking a “Human memory and knowledge test.” Then, after reading and signing the consent form, participants were given the Word-pair learning task first, followed by the cognitive reflection test. Both are described in more detail below.

##### 3.1.3.1. Word-pair learning task

Participants went through two blocks of the pair task. Each block consisted of studying and making a judgment of learning for a series of 30 word-pairs. Each study presentation lasted for 5 s for each pair and JOLs were made using the Likert scale (0, 20, 40, 60, 80, and 100%). After studying and rating the entire list, participants were given a cued-recall test, having to type in the second word given the first, e.g., “chair—()”. When the first block was completed, a second block began.

##### 3.1.3.2. Cognitive reflection test

After two blocks of the word-pair learning task, participants solved seven CRT questions. The questions consisted of problems to solve, for example, “A bat and a ball cost $1.10 in total. The bat costs a dollar more than the ball. How much does the ball cost?” At first glance, if participants were to think intuitively, they might answer “10 cents,” but that would be incorrect. However, given more time and deliberation, they would be more likely to come up with the correct answer “5 cents.” Before beginning the CRT task, we asked participants if they were familiar with the CRT. If they were, those participants’ data were excluded from the analysis.

#### 3.1.4. Results

##### 3.1.4.1. Calculating the metacognitive monitoring index

The most common method in which to measure metacognitive monitoring is to record an individual’s confidence. When using ratings, there are two different times at which to record confidence (Nelson, 1990). The first, already introduced above, is a prospective judgment, which is one that has individuals giving a rating during learning, but *prior to test*, known as the JOL. Another type of rating is retrospective confidence (RC) judgment, which rates, *after test*, the probability of being correct (Glenberg and Epstein, 1985). This method is generally used in educational studies (Dunlosky and Nelson, 1992, 1994), but is also used as a method of measuring confidence to confirm metacognitive monitoring in various research fields—decision making, problem-solving and even athletics (Nietfeld, 2003; Rozencwajg, 2003; Wokke et al., 2017; Bellon et al., 2020). Very simply, after giving an answer to some problem, one’s RC would be the answer to the subsequent question, “How certain are you in your answer?” Scientifically, among various methods of measurement, there is one formula that has been shown to be fairly easy to use (Schraw, 2009), representing what is known in metacognition as the *absolute accuracy index* (Keren, 1991; Nelson et al., 2004; Allwood et al., 2005; Burson et al., 2006). Absolute accuracy assesses the precision of the confidence judgment when compared to actual performance on a task (Maki et al., 2005). The most common measure of absolute accuracy is shown in formula (1):

(1) $$\mathrm{Absolute Accuracy Index}=\frac{1}{N}\sum_{i=1}^{N}{\left(c_{i}-p_{i}\right)}^{2}$$

Here, $c_{i}$ corresponds to a retrospective confidence rating $p_{i}$ which corresponds to a performance score, while *N* corresponds to the number of items. This is also referred to as the *calibration index*. The lower the index (closed to zero), the more accurate is one’s metacognitive monitoring accuracy.

If the correct answer rate for any specific question was too high, that question was excluded. What remained were problems with similar success rates (see Supplementary Table 1 *for accuracy rates across items*)—the within-subject Analysis of variance (ANOVA) comparing the accuracy among each CRT item showed no differences.

Each individual score for monitoring ability was calculated by using their absolute accuracy (AA) index. We then calculated a Pearson correlation between AA and CRT accuracy, and found a significant negative correlation between the two (*r* = −0.33, *N* = 100, *p* < 0.001), suggesting that the lower (more accurate) one’s monitoring accuracy index, the higher one’s CRT accuracy. We then divided the subjects into high/low monitoring ability groups by using the median of the AA, median = 0.0905 as the boundary (Maki and Berry, 1984; Thiede et al., 2003; Moreno and Saldaña, 2005). Results showed a significant difference in CRT accuracy for those in the high monitoring ability group (*N* = 50, *M* = 0.52, SD = 0.19) versus those in the low monitoring ability group (*N* = 50, *M* = 0.43, SD = 0.22) group [*t*(98) = 2.16, *p* < 0.05]. A *t*-test, however, showed no significant differences in accuracy for the word-pair memory task between the High (*N* = 50, *M* = 0.65, SD = 0.17) and Low (*N* = 50, *M* = 0.61, SD = 0.16) groups [*t*(98) = 0.90, *p* = 0.37], suggesting that there was no confounding between CRT and memory.

#### 3.1.5. Discussion

In Experiment 1, we found that people who have relatively higher metacognitive monitoring ability were more successful at problem solving on the CRT task. No difference was found on the memory task. Based on these results, we were then ready to conduct Experiment 2 to explore further how monitoring abilities are affected when problem-solving. In particular, we asked whether positive/negative emotions would affect monitoring accuracy in different ways, and hypothesized that, ironically, those high in metacognitive monitoring might be more likely to use up their cognitive resources, thus resulting in fewer successfully solved problems.

## 4. Experiment 2

Experiment 2 was conducted to investigate whether emotions, particularly negative emotions, would affect people’s problem-solving ability as well as the interaction, if any, of one’s metacognitive monitoring ability. First, we wanted to test the notion that an individual with higher metacognitive ability might, surprisingly, be more influenced by negative emotions, given more depletion of cognitive resources. Overall, then, we hypothesized the following. When in a positive emotional state, those with high metacognitive ability may demonstrate higher CRT accuracy as compared with those lower in metacognitive ability, given that positive emotions might not require a ton of resources. However, when in a negative emotional state, those with high metacognitive ability would show similar disruptions, or perhaps more, in CRT accuracy, when compared to those with low metacognitive ability. This would mean that those high in sensitivity to emotional states, or *high monitors*, may be using up the resources that are needed in the moment during problem solving.

Prior to completing the CRT, participants’ emotional states were induced by showing videos—positive or negative. As before, we then calculated the Absolute Accuracy Index to check participants’ overall monitoring abilities. Then, as an additional extension to Experiment 1, we also asked participants whether or not they wanted to see the problems again after completing the CRT problems. This was to examine whether their metacognitive *control* processes would be affected. For instance, requesting to see the problems again if they were incorrect, but not correct, would be an example of appropriate control. On the other hand, if they asked to see the problems again after they were correct, or randomly, that may be an indication of sub-optimal control (Dunlosky and Metcalfe, 2008). Including this repetition request procedure would allow us to pull apart the two processes of metacognition, monitoring and control, and to see the effects of emotion. Finally, as in Experiment 1, both the Word-Pair Learning Task and the CRT were included given that metacognition has domain-general features (Bellon et al., 2020).

### 4.1. Method

#### 4.1.1. Participants

A total of 104 undergraduate students $(M_{age}=22.95$, $N_{male}=46$, $N_{female}=58)$ attending Ajou University participated in Experiment 2. None had participated in Experiment 1. We used data from 102 participants to analyze because two of the participants’ Absolute Accuracy Index fell right on the median. All participants signed consent forms prior to the experiment and received credits or gift cards for their participation.

#### 4.1.2. Materials

The word pair learning task and CRT were used as had been in Experiment 1. Added in Experiment 2 were the emotion manipulation videos, described below.

##### 4.1.2.1. Emotion manipulation video

The video consisted of two bundles of video or movie clips (ranging from 5 min 15 s to 6 min 45 s) to induce positive or negative emotions (Supplementary Table 2). These bundles were made up of eight clips from 32 videos or movies, which were selected from the highest scores for each emotion in a pilot test. The pilot check was based on a procedure from Jang et al. (2005), and used a series of descriptions—two positive (joyful, relief) and two negative (sadness, fear)—that were rated on a 7-point Likert scale for agreement. One question—that rated how much “overall emotion” was experienced, was rated on an 11-point scale. Results showed no differences in the amplitude of the negative and positive induced emotional states $(M_{negative}=8.80,SD_{negative}=1.77,M_{positive}=8.70,SD_{positive}=1.53$, *t* = 0.30, *p* = 0.77). In other words, participants felt both the positive and negative emotions with similar power. We conducted paired sample t-test between the positive and negative emotion scores (Positive video clip *M difference < positive–negative score >* =7.29, SD = 2.20, *t* = 23.90, *p* < 0.001; Negative video clip *M difference < positive–negative score >* = − 7.01, SD = 2.60, *t* = −20.22, *p* < 0.001). The resulting videos were eight in total—four positive videos and four negative videos. These represented the two videos in each of the categories that received the highest and second-highest emotion ratings. That is, we had the top two videos rated for *joy*, the top two videos rated for *relief*, the top two videos rated for *sadness*, and the top two videos rated for *fear*.

#### 4.1.3. Procedure

In general, the procedure was the same as that of Experiment 1. The crucial addition was the presentation of the video clips for the emotion manipulation, and the secondary addition was the repetition request section as a way to examine one’s metacognitive control processes.

As before, to measure metacognitive ability, participants were first presented with two blocks of the word-pair learning task. In addition, however, during the cued recall test, after providing their answer to each cue, to check their control decisions, they had the opportunity to choose whether to re-answer the cue prompt or to go on to the next cue. After the second block, participants, randomly assigned to either negative or positive emotion video clips, were presented with two video clips, counter-balanced for order. For instance, one participant in the positive condition might have been presented with the video that rated highest on “relief” followed by the video that was rated highest on “joy;” another participant in the positive condition might have been presented with the video that was rated highest for joy and second-highest for relief, and so on. In the instructions, participants were told the following: “You will be watching a video with a series of clips in order to prevent any confounding after end of second block” before showing the video clip.

**Figure 1:**
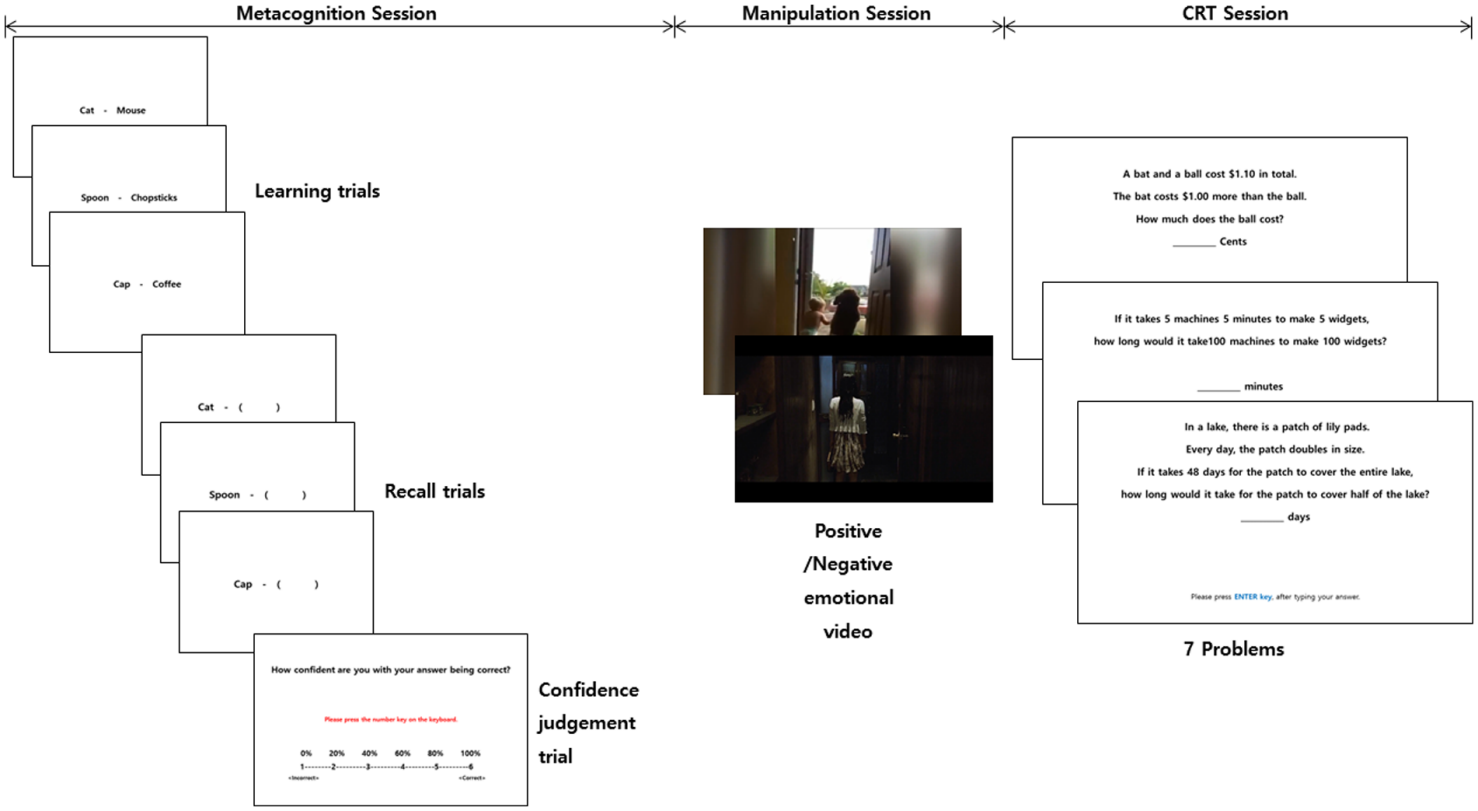
Detail procedure in Experiment 2.

After watching the video clips, they immediately performed the CRT (Figure 1). In this session, participants saw each CRT problems for 5 s first. They then had 5 min to solve and rate their confidence for those problems. After rating their confidence, they then had the opportunity to choose whether to solve the problem again or go onto the next CRT problem. That is, participants were allowed repeated attempts to solve the problem, and were told that they could simply move on to the next problem if they felt they could not solve it. *Control* was coded as zero if participants did well, and one if they did not (contrary to what might be intuitive). And note that we coded a 0 (good control) for two scenarios: One, when participants solved the problem correctly after they re-tried to solve the problem, or two, when they were unable to solve the problem after passing on re-trying to solve the problem. These define “good control” as people “know what they could know” and “know when they will not know.”

Finally, we conducted a survey to check the success of the emotional manipulation (see the questionnaire form Supplementary Figure 2; Jang et al., 2005). This form aimed to rate, also on a 7-point scale, what kind of emotions and how strongly participants felt the emotion while watching the videos. On the survey, participants also had room to express whatever emotion they had, in case they felt mixed emotions.

#### 4.1.4. Design

**Figure 2:**
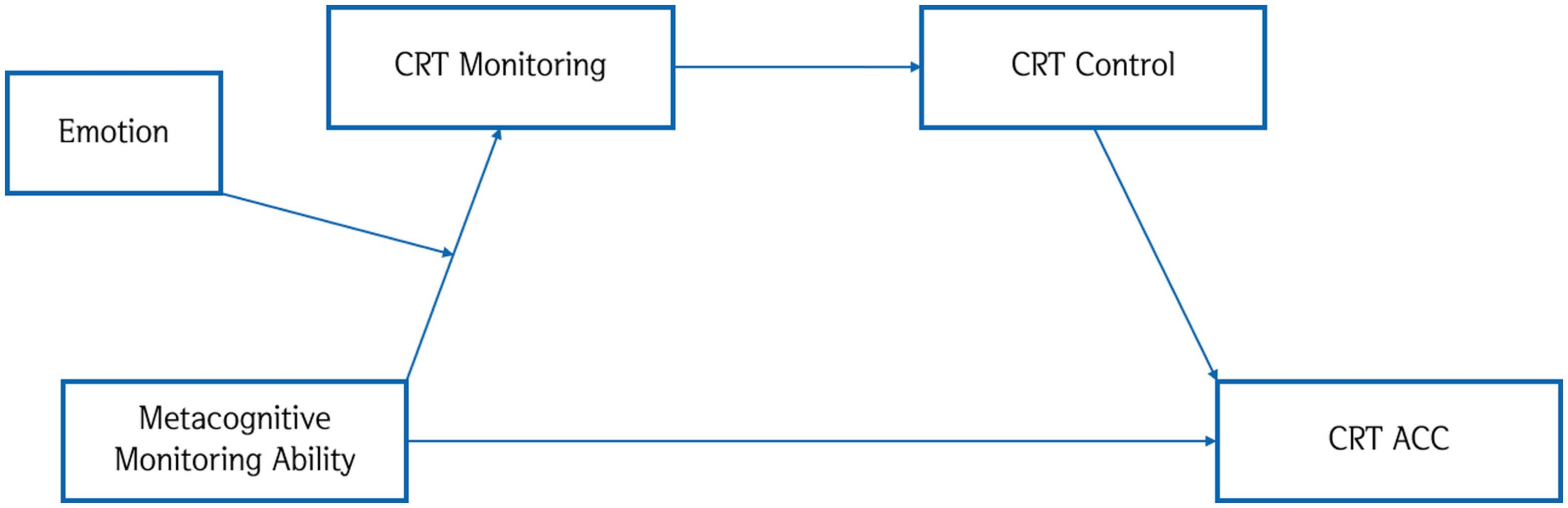
Hypothetical model.

We hypothesized that negative emotions would moderate metacognitive monitoring ability, resulting in poorer CRT monitoring. As a consequence, control functions would be impacted negatively as well. Put simply, those in the negative emotion condition would be overall worse at solving the CRT items as compared to those in the positive emotion condition. Figure 2 presents our hypothetical model.

Before confirming the moderated mediation model, we designed a two (Metacognitive monitoring ability group: High vs. Low) × 2 (Emotions: Negative vs. Positive) framework where a two-factorial design between participants was conducted. Subjects were placed into high-meta and low-meta groups by using the median of the Absolute Accuracy (AA median = 0.080), in the same way as was done in Experiment 1 (Equation 1). Thus, if a subject’s AA was lower than 0.080, the subject was placed in the high monitoring ability group; and if the Absolute Accuracy was higher than 0.080, the subject was placed in the low monitoring ability group (to note, higher scores represent worse AA). Two participants were excluded from the analysis because their Absolute Accuracy fell right on the median and therefore could not be assigned to either the high or the low-meta monitoring groups.

#### 4.1.5. Results

To check the manipulation, we first conducted t-tests to confirm the differences in positive and negative emotion conditions. The descriptive statistics can be found in Supplementary Table 3. Our results showed a successful manipulation as those who watched the positive video gave significantly higher positive emotion ratings [*t*(102) = −20.11, *p* < 0.001] while those who watched the negative video gave significantly higher negative ratings [*t*(102) = 18.72, *p* < 0.001].

**Figure 3:**
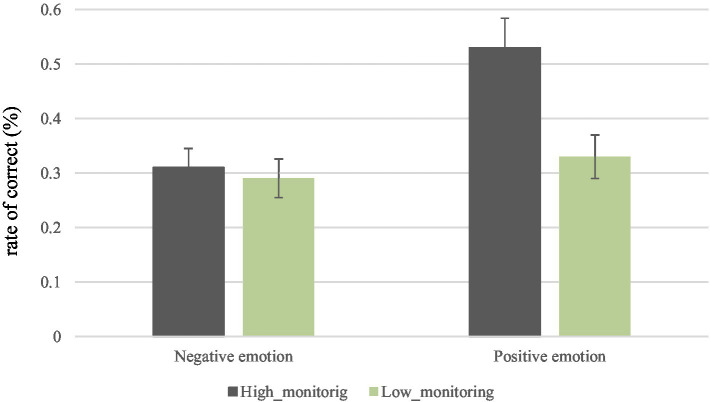
Accuracy rate of cognitive reflection test (CRT) between high/low metacognitive monitoring ability groups and emotion manipulation in Experiment 2.

We then turned towards the metacognitive monitoring and control abilities and their effects on CRT performance. First, we conducted a two-way ANOVA to confirm the interaction effects of monitoring level on CRT accuracy. The descriptive statistics (Table 2) and the interaction graphs between variables are presented in Figure 3. We divided high and low monitoring group by splitting at the median (median = 0.08), representing monitoring ability. Two participants were excluded from the analysis because their scores fell right on the median value. The ANOVA resulted in a significant interaction between metacognitive monitoring ability and emotion manipulation [*F*(1, 98) = 4.59, *p* < 0.05], as well as significant main effects of emotion manipulation [*F*(1, 98) = 9.62, *p* < 0.01] and metacognitive monitoring ability [*F*(1, 98) = 7.12, *p* < 0.01].

**Table 2:** Descriptive statistics of cognitive reflection test (CRT) accuracy between the high/low metacognitive monitoring ability groups and emotion manipulation in Experiment 2.

	Low-monitoring			High-monitoring			Total		
	*N*	*M*	SD	*N*	*M*	SD	*N*	*M*	SD
Negative emotion	26	0.29	0.18	26	0.31	0.18	52	0.30	0.18
Positive emotion	25	0.33	0.20	25	0.53	0.27	50	0.43	0.26
Total	51	0.31	0.19	51	0.41	0.25	102	0.36	0.23

Given the interaction, we also conducted simple-comparison analyses. Results showed a significant difference between emotion in the high-monitoring $(M\;difference_{\langle negative-positive\rangle }=-.22$ SD = 0.06, *p* < 0.001), but not in low-monitoring $(M\;difference_{\langle negative-positive\rangle }$= − 0.04, SD = 0.06, *p* = 0.50) ability group. In other words, only those participants who were relatively better at monitoring seemed to be influenced by the emotional manipulation; those relatively poorer at monitoring were not. Also, there was a significant difference between monitoring groups for the positive emotion manipulation $(M\;difference_{\langle high-low\rangle }$=0.20, SD = 0.06, *p* < 0.001), supporting the results from Experiment 1. However, there were no significant differences between monitoring ability groups for the negative emotion manipulation $(M\;difference_{\langle high-low\rangle }$=0.02, SD = 0.06, *p* = 0.71), hinting that the negative emotion may have affected the accuracy rate of those participants who were “good” at monitoring, decreasing their performance to the point where it matched the performance of those who were “poorer” monitors.

**Figure 4:**
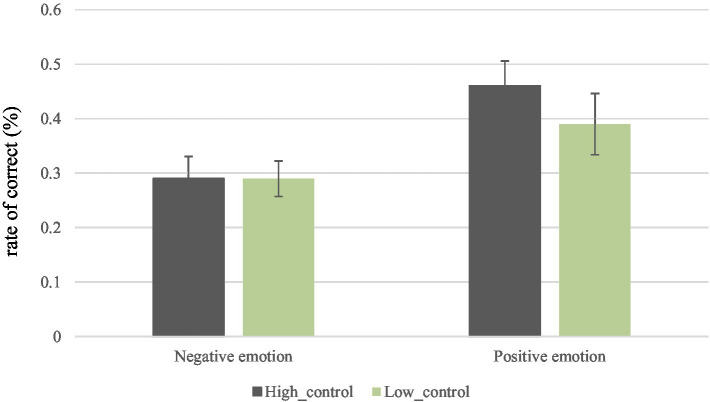
Correct rate of cognitive reflection test (CRT) between high/low level of metacognitive control ability groups and emotion manipulation in Experiment 2.

We then investigated the relationship between metacognitive control ability and CRT accuracy using the same method as above. Table 3; Figure 4 display the results. Participants were divided into high and low control groups in same way as had been done with the monitoring ability groups above (median = 0.367, excluding five participants, as they fell right on the median score). Results showed no significant interaction [*F*(1, 95) = 0.82, *p* = 0.37] for control ability and emotion manipulation, and no differences in control ability [*F*(1, 95) = 0.62, *p* = 0.44]. However, the emotion manipulation resulted in a main effect [*F*(1, 95) = 9.11, *p* < 0.01]. After conducting simple comparisons to uncover which variables drove the effect, we found a significant difference between the emotion manipulation conditions for the high-metacognitive control ability group $(M\;difference_{\langle negative-positive\rangle }$= − 0.18, SD = 0.06, *p* < 0.01), but not for the low control ability group $(M\;difference_{\langle negative-positive\rangle }$= − 0.09, SD = 0.06, *p* = 0.14). In other words, participants who were “originally better” at control seemed more affected by the emotion manipulation than the participants who were “originally poorer” at control.

**Table 3:** Descriptive statistics of cognitive reflection test (CRT) accuracy between the metacognitive control ability groups and emotion manipulation in Experiment 2.

	Low-control			High-control			Total		
	*N*	*M*	SD	*N*	*M*	SD	*N*	*M*	SD
Negative emotion	27	0.29	0.17	22	0.29	0.19	49	0.29	0.18
Positive emotion	23	0.39	0.27	27	0.46	0.24	50	0.43	0.26
Total	50	0.33	0.22	49	0.38	0.23	99	0.36	0.23

Also, both positive $(M\;difference_{\langle high-low\rangle }$=0.08, SD = 0.06, *p* = 0.23) and negative emotion groups (*M difference*_<*high*-*low*>_=0.01, SD = 0.06, *p* = 0.93) revealed no differences between participants in the two different metacognitive control ability groups. To put it simply, only the high metacognitive control ability group was “affected” by the emotion manipulation.

##### 4.1.5.1. Regression analysis

According to above results, we found that accuracy on the CRT task was affected by both monitoring and control abilities. In addition, we found that emotion disproportionately affected those higher in metacognitive processes. Therefore, we then used the PROCESS Procedure for SPSS (version 3.5; Hayes, 2017) to investigate a moderate mediation of emotion model to evaluate our hypothesis model against a competitive model. The reason we set up the competitive model was because prior emotion studies had shown that positive and negative emotions affected problem-solving differently (Isen et al., 1985, 1987; Hirt et al., 1996; Crooks and Alibali, 2013). First, we focused on the monitoring component (Supplementary Figures 5, 6). This method incorporated a bootstrap approach (Baron and Kenny, 1986) to estimate the indirect effects. Descriptive statistics and correlational results are presented (Supplementary Table 6) as well as the results of the regression and indirect effects (Supplementary Table 7).

The results showed that monitoring ability (Meta M) interacted with emotion to affect CRT monitoring (*b* = −2.58, SE = 0.67, *p* < 0.001). And there was a significant association in both Meta M (*b* = 1.89, SE = 0.52, *p* < 0.001) and emotion (*b* = 0.35, SE = 0.07, *p* < 0.001) to the CRT monitoring path. Furthermore, there was a significantly associated path from CRT monitoring to CRT accuracy (*b* = −0.47, SE = 0.10, *p* < 0.001) as well as a path from Meta M to CRT accuracy (*b* = −0.75, SE = 0.38, *p* < 0.05). The results from the bootstrapping procedures showed that the 95% confidence interval around the indirect effects did not contain a zero (0.52, 2.88), indicating that the mediation effect was significant. We also found a moderation of emotion on the mediation effect. As shown in Supplementary Table 8, the conditional mediation effect was significant for the positive emotion manipulation [*b* = −0.89, SE = 0.50, 95% CI = (−2.38, −0.41)].

Then, to compare the effect of emotion on the path from Meta M to CRT accuracy, we conducted the same analysis again (Supplementary Figure 6), and confirmed no significant effect of a Meta M interaction (*b* = −0.06, SE = 0.82, *p* = 0.95). That is, Meta M may affect emotion, but does not affect CRT accuracy. This result suggests that the emotion manipulation affected CRT monitoring, which subsequently affected CRT accuracy, but indirectly, and not, rather, on the direct path from Meta M to CRT accuracy.

We also ran models for control ability (Meta C). We conducted these models in the same way that we ran the models for monitoring. There are two models for comparison (Supplementary Figures 7, 8). In model 3 (Supplementary Table 12), there was a significant association in both Meta C (*b* = 0.67, SE = 0.16, *p* < 0.001) and emotion manipulation (*b* = 0.56, SE = 0.09, *p* < 0.001) with CRT control. Meta C interacted with emotion, affecting CRT control (*b* = −0.86, SE = 0.22, *p* < 0.001). CRT accuracy was affected solely by CRT control (*b* = −0.38, SE = 0.08, *p* < 0.001), and not by Meta C (*b* = 0.01, SE = 0.11, *p* = 0.93). The results from the bootstrapping procedures showed that the 95% confidence interval around the indirect effect did not contain a zero (0.15, 0.58), indicating that the mediation effect was significant. We also found a moderation of emotion on the mediation effect. As shown in Supplementary Table 13, the results showed a significant conditional mediation effect for the positive emotion manipulation [*b* = −0.26, SE = 0.09, 95% CI = (−0.46, −0.12)].

We next ran model 4 (Supplementary Figure 8), which was to confirm any effects of emotion on the direct path from Meta C to CRT accuracy. The results are shown in Supplementary Tables 18, 19. As can be seen, there was no association among Meta C (*b* = −0.01, SE = 0.19, *p* = 0.97) and emotion (*b* = 0.18, SE = 0.12, *p* = 0.15), but Meta C interacted with emotion (*b* = −0.51, SE = 0.27, *p* = 0.06) with CRT accuracy. Model 4 was also the same as model 2.

These results suggest that the participants’ metacognitive abilities were affected by the emotions when solving CRT problems, and, consequently, emotions also affected their CRT accuracy. Stemming from the result above, we thought that monitoring ability would affect emotion, and, thus, control ability as well as CRT accuracy. Therefore, we checked these paths as well, conducting the PROCESS macro again to verify our hypothesis model (see Figure 2), using moderated serial mediation. Descriptive statistics are presented (Supplementary Table 16) for model 5 and 6, as well as regression results for model 5 (Supplementary Table 17) and model 6 (Supplementary Table 18). The results showed that CRT monitoring was significantly affected by metacognitive monitoring ability (Meta M, *b* = 1.89, *p* < 0.001) and emotion (*b* = 0.35, *p* < 0.001). And we were able to see that Meta M interacted with emotion and CRT monitoring (*b* = −2.58, *p* < 0.001). Also, CRT control was affected by only CRT monitoring (*b* = 0.60, *p* < 0.001), not by Meta M (*b* = 0.72, *p* = 0.09). We also found indirect effects in model 5. The results from the bootstrapping procedures showed that the 95% confidence interval around the indirect effect did not contain a zero (0.07, 1.00), indicating that the mediation effect was significant. As shown in Supplementary Table 19 (see model 5 raw), the results presented that conditional mediation effect was significant for positive emotion [*b* = −0.27, SE = 0.19, 95% CI = (−0.81, −0.06)].

And, to compare with five, we conducted the same analysis with one path added between Meta M interacted by emotion and CRT control (model 6). The only difference from model 5 was the path from Meta M to CRT control. There were significant differences among Meta M (*b* = 2.41, *p* < 0.001), emotion (*b* = 0.45, *p* < 0.001), CRT monitoring (*b* = 0.31, *p* < 0.01), and Meta M interacted by emotion (*b* = −2.78, *p* < 0.001). Also, there was an indirect effect (0.00, 0.51), and a conditional mediation effect at emotion [*b* = −0.14, SE = 0.10, 95% CI = (−0.39, −0.00)].

To get an idea of which portion of one’s metacognitive abilities was affected by emotion, we conducted a two-way ANOVA to check if there had been a difference in metacognitive monitoring ability before and after the emotion manipulation (see Supplementary Table 4; Figure 3). We were able to confirm an interaction effect between metacognitive monitoring ability and emotion manipulation [*F*(1, 98) = 21.87, *p* < 0.001], and significant main effects for both emotion manipulation [*F*(1, 98) = 17.49, *p* < 0.001] and metacognitive monitoring ability [*F*(1,98) = 12.49, *p* < 0.001]. Using simple-comparison analysis for each independent variable, we found that for the high-monitoring ability group, there was a significant difference between emotion manipulation conditions (*M difference < negative–positive >* =0.29, SD = 0.05, *p* < 0.001). None was found for the low-monitoring ability group (*M difference < negative–positive >* = − 0.02, SD = 0.05, *p* = 0.73). In other words, the pattern suggests that the negative emotion affected monitoring ability, but especially for the high-monitoring ability group.

Finally, we conducted the same analyses for control ability. Supplementary Table 5; Figure 4 presents the results. There was an interaction effect between metacognitive control ability and emotion for CRT control [*F*(1, 95) = 24.38, *p* < 0.001] as well as the significant main effects in emotion [*F*(1, 95) = 34.27, *p* < 0.001], but no effect on metacognitive control ability [*F*(1, 95) = 3.49, *p* = 0.07]. We checked each independent variable, using simple-comparison analysis, and found a significant difference between emotion in the high-control ability group (*M difference < negative–positive >* =0.44, SD = 0.06, *p* < 0.001) but, not in the low group (*M difference < negative–positive >* =0.04, SD = 0.06, *p* = 0.52). The differences between control ability groups were significant different for positive emotion (*M difference < high-low >* = − 0.28, SD = 0.06, *p* < 0.001) and negative emotion (*M difference < high-low >* =0.13, SD = 0.06, *p* < 0.05). Thus, overall, we found that control ability was affected by the emotion manipulation, namely for the high-control ability group.

## 5. General discussion

This study tested whether emotion affected monitoring and control abilities in different ways. The data show that, indeed, negative emotion disproportionately impacted those higher in monitoring and control, suggesting that metacognition during problem solving employs the same resources that are taken away under negative emotional states. These results present a novel scenario in which those who possess stronger, or more sensitive, levels of metacognition could be at a disadvantage, as compared to those with poorer, or less sensitive levels of metacognition. Given the ambiguity in the past literature, the present data allows for an interpretation that suggests a resource-depletion view for high monitors as compared to low monitors.

In Experiment 1, the cognitive reflection test was administered to benchmark the relationship between one’s general monitoring ability and problem-solving strategies. Results showed that, as expected, participants with high-monitoring abilities used a greater number of appropriate strategies to solve the problems. In Experiment 2, we sought to understand if there was a change in the use of those problem-solving strategies when put into a particular emotional state, and whether those changes would affect individuals differently based on their monitoring abilities. We had hypothesized that, somewhat ironically, more of the cognitive resources needed to process negative emotion would be more damaging for individuals with high metacognitive monitoring abilities, as they are already using relevant resources. Results supported our hypothesis. Negative emotions not only disrupted problem solving more than positive resources, but the negative emotions also disrupted those who had stronger, or more sensitive monitoring levels.

In Experiment 2, using moderate serial mediation of and emotion model, we were able to obtain a clearer picture of how emotional states might affect monitoring and control abilities of individuals. In line with the earlier results, we found that those who are stronger in control abilities also seem more negatively impacted by the negative emotional states. That the better monitoring and control abilities people have, the more influenced by emotion they are. Taken together, the results of the current study show that both the monitoring and control processes of metacognition are affected by emotion states, and, ironically, those who are generally stronger in metacognition, receive more of the brunt of the disruption.

Previous studies have shown that subjects who have high monitoring ability may be more sensitive to negative emotions, leading to the use of effective strategies to escape negative emotional states, and leading to reduced negative emotions (Harris et al., 1981; McCoy and Masters, 1985; Davis et al., 2010; Nguyen et al., 2018). However, in situations where negative emotions occur or immediately after they occur, some number of cognitive resources are allocated to emotional processing, which may take them away from finding appropriate strategies for problem-solving. As a result, performance for those with already low monitoring abilities may drop further. This was not the case here. The steeper drop for those with higher monitoring abilities was the critical result.

We also discovered that the metacognitive monitoring ability interacted with the emotion-affected metacognitive control ability (see the path from Meta M*emo ➔ CRT control in Supplementary Table 18). This effect was a result of not applying sufficient control processes when solving the CRT problems, again, because, presumably, the cognitive resources were “taken away” to process the negative emotions (Nguyen et al., 2018). In future studies, it would help to see if these effects persist over longer periods of time. For example, maybe there is a flip—perhaps those with higher basic levels of metacognition only temporarily are disadvantaged by the negative emotion, but in the longer run, are able to get some control over both the emotion and the problem-solving task.

This study is meaningful in that it suggests that the influence of emotion is complex. And how metacognitive resources are allocated and applied are not yet entirely understood. We already know that those with good metacognitive skills may over- or underestimate their performance when put in a stressful situation (Spada et al., 2008). As a result, they may try to solve an unsolvable situation or give up prematurely (as we have shown in Supplementary Table 20). At the same time, metacognitive sensitivity is enormously important for all kinds of other problem-solving tasks. That is, we are not advocating for people to think of metacognition as a hindrance. Instead, we would support a program that decreases a negative emotion environment, as much as can be done. Or, in the least, have people understand the interference that negative emotions bring (Li and Roloff, 2006; Van Kleef, 2008; Campo et al., 2016; Kopp and Jekauc, 2018). One simple example can be seen in Li and Roloff (2006), where they mention that if high monitoring people feel negative emotions in a negotiation situation, they would be wise to hold off, slow down, and give themselves an opportunity to get resources back.

In summary, based on the results of this study, we can conclude that the “best metacognizers” might find themselves in situations that hurt their ability to solve a problem “the most.” Namely, when negative emotions arise, the resources that they typically need to monitor and control their strategies are disrupted more than if they were “weaker metacognizers” to begin with. Given these results, we can suggest that problem solving is vulnerable, and thinking about the context in which you approach a problem should not be ignored. Some of the limitations of our study are that we cannot yet say how long the effects persist. According to Basso et al. (2019), for instance, people usually take about 13 min to lessen the negative emotion. Perhaps it would depend on overall monitoring ability as well. This question of time, in addition to other factors that have yet to be determined, are essential to understanding the path to success during problem solving. In the least, we have been excited to show that monitoring may be swayed by one’s emotional state, and from an empirical perspective, we look forward to further investigations into the impact that emotion has on both the monitoring and control processes, and in the short term and the longer term.

## Data availability statement

The raw data supporting the conclusions of this article will be made available by the authors, without undue reservation.

## Ethics statement

The studies involving human participants were reviewed and approved by Institutional Review Board, Ajou University (202003-HB-001). Written informed consent to participate in this study was provided by the participants’ legal guardian/next of kin.

## Author contributions

S-sH: study design, data collection, analysis, management, manuscript writing, and review and editing. JB: analysis and manuscript drafting review & editing. LS and KK: manuscript writing, review and editing. All authors contributed to the article and approved the submitted version.

## Funding

This work was supported by the Ministry of Education of the Republic of Korea and the National Research Foundation of Korea (NRF-2022S1A5B5A16052792).

## Conflict of interest

The authors declare that the research was conducted in the absence of any commercial or financial relationships that could be construed as a potential conflict of interest.

## Publisher’s note

All claims expressed in this article are solely those of the authors and do not necessarily represent those of their affiliated organizations, or those of the publisher, the editors and the reviewers. Any product that may be evaluated in this article, or claim that may be made by its manufacturer, is not guaranteed or endorsed by the publisher.
