# Supplementary material for: Negative emotion can be “more negative” for those with high metacognitive abilities when problem-solving
Source: Front Psychol. 2023 Mar 13;14:1110211. doi: 10.3389/fpsyg.2023.1110211 (PMC10043754; doi:10.3389/fpsyg.2023.1110211)
Supplement: Supplementary file 1 [file Data_Sheet_1.docx]

Supplementary Material

# Supplementary Figures and Tables

## Supplementary Figures

##
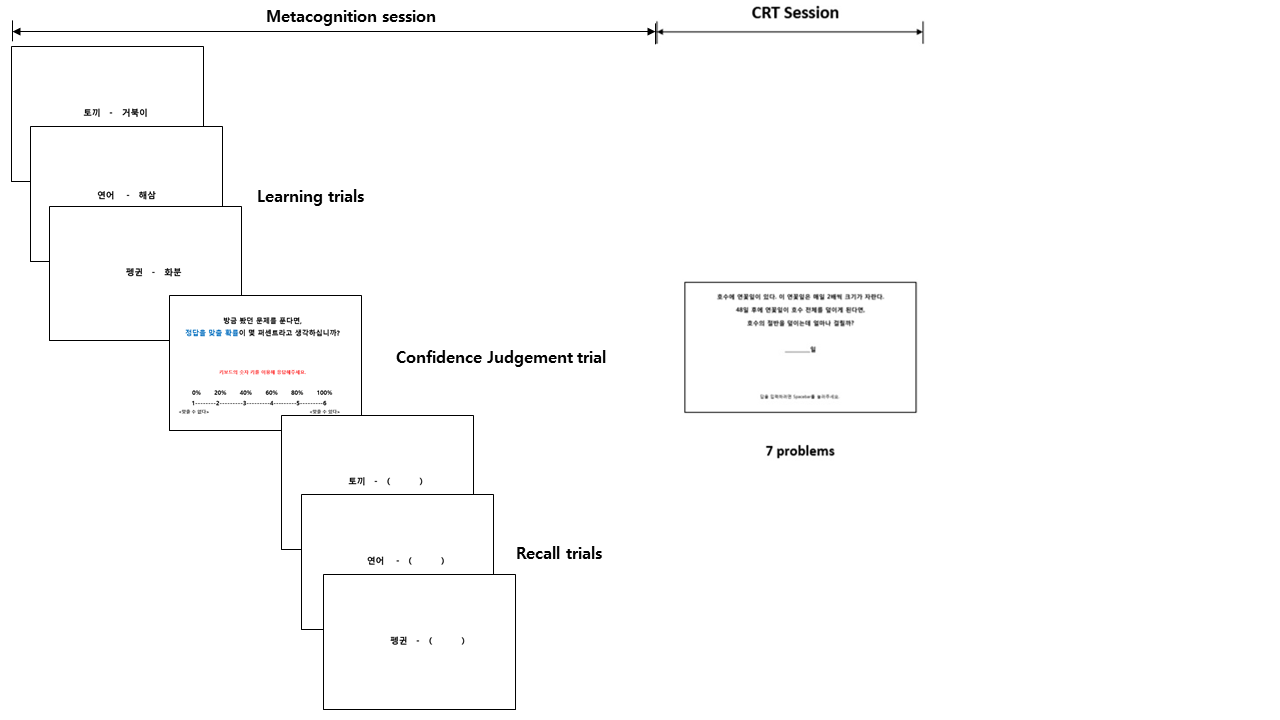


Supplementary Figure 1. Detail procedure in Experiment 1.


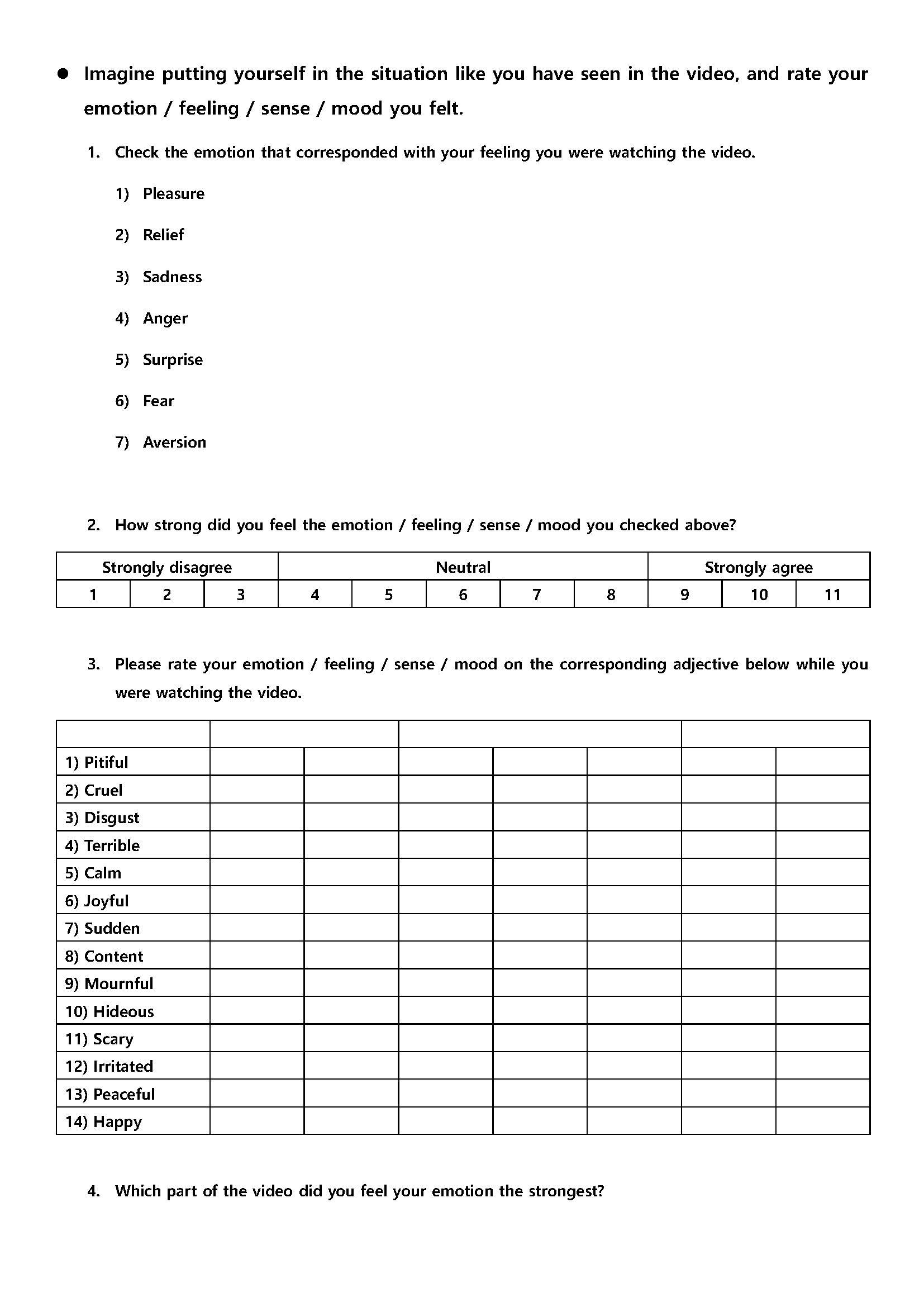
Supplementary Figure 2. Korean / English version survey form to rate induced emotion.

Supplementary Figure 3. CRT monitoring between high/low level of metacognitive monitoring ability groups and emotion manipulation in Experiment 2.

Supplementary Figure 4. CRT control between high/low level of metacognitive control ability groups and emotion manipulation in Experiment 2.


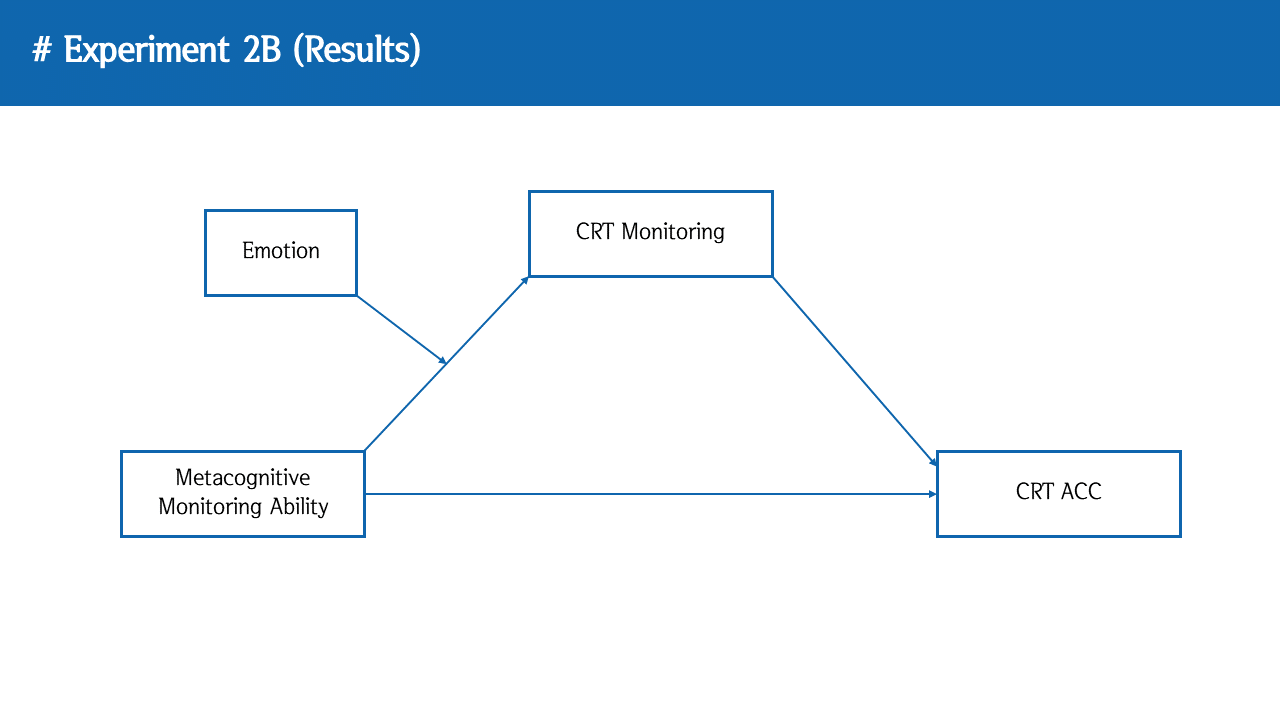


Supplementary Figure 5. Moderate mediation of emotion model 1 for monitoring.


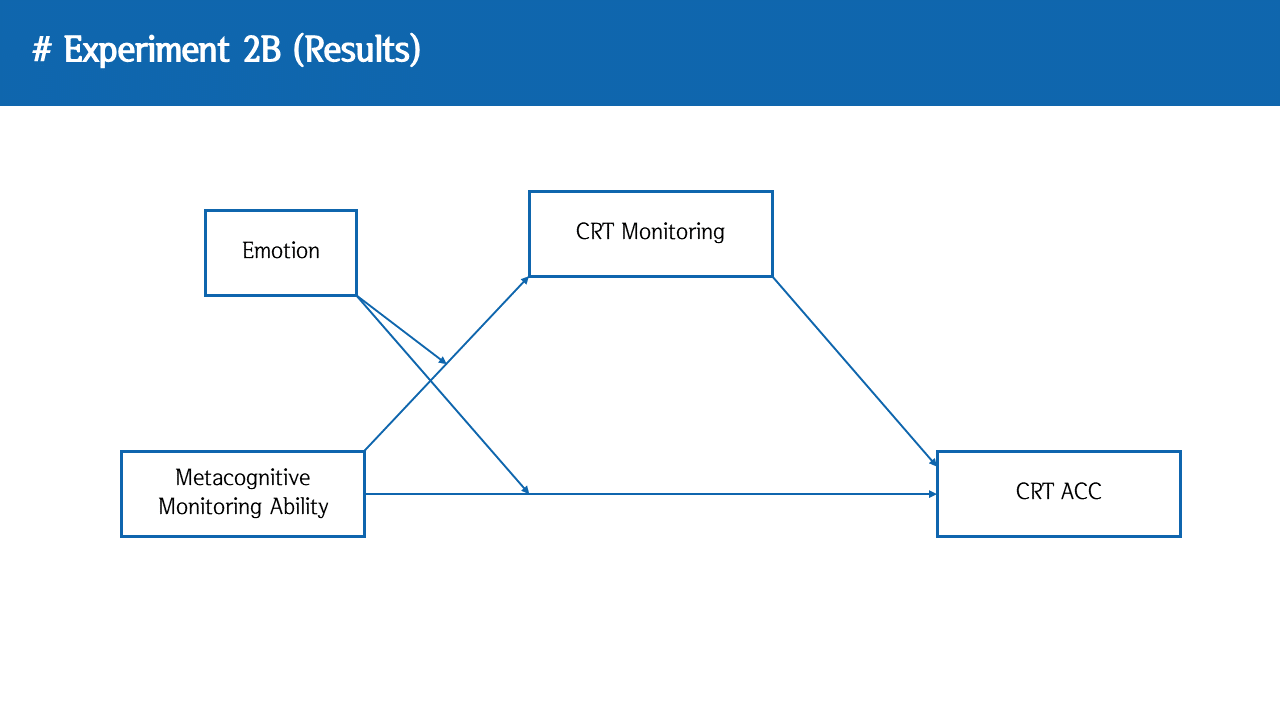


Supplementary Figure 6. Moderate mediation of emotion model 2 for monitoring.


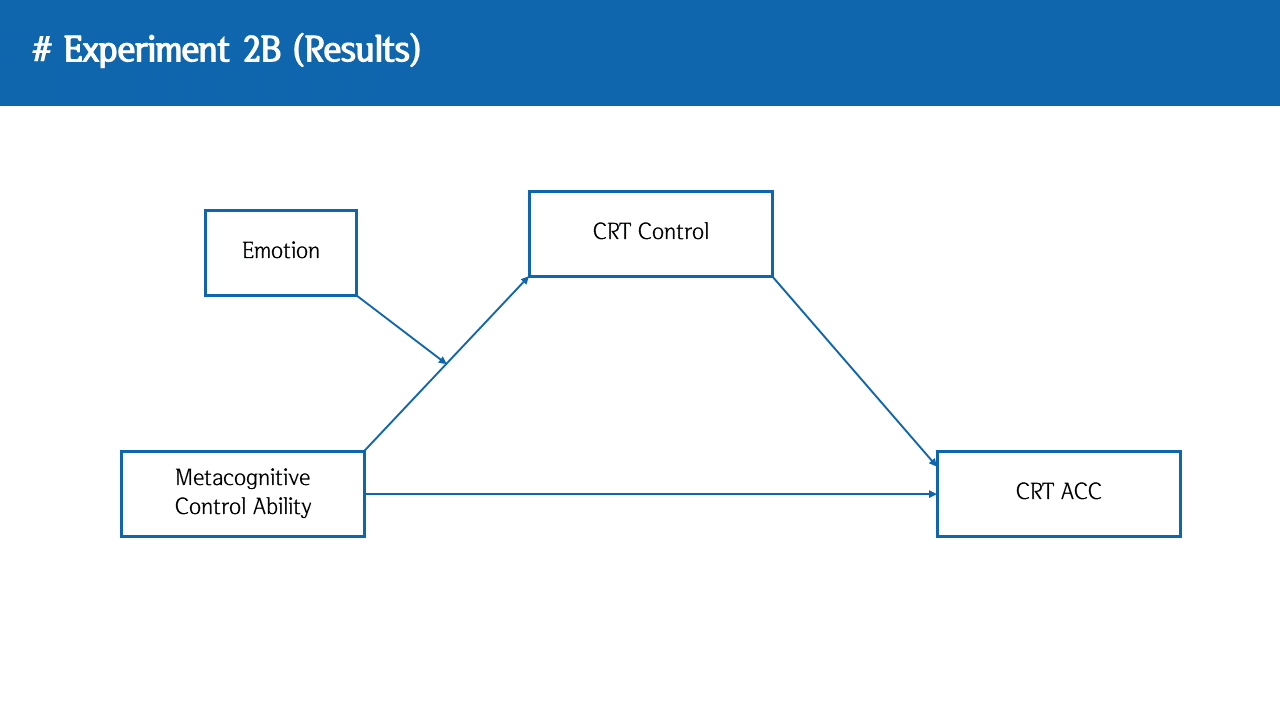


Supplementary Figure 7. Moderate mediation of emotion model 3 for control.


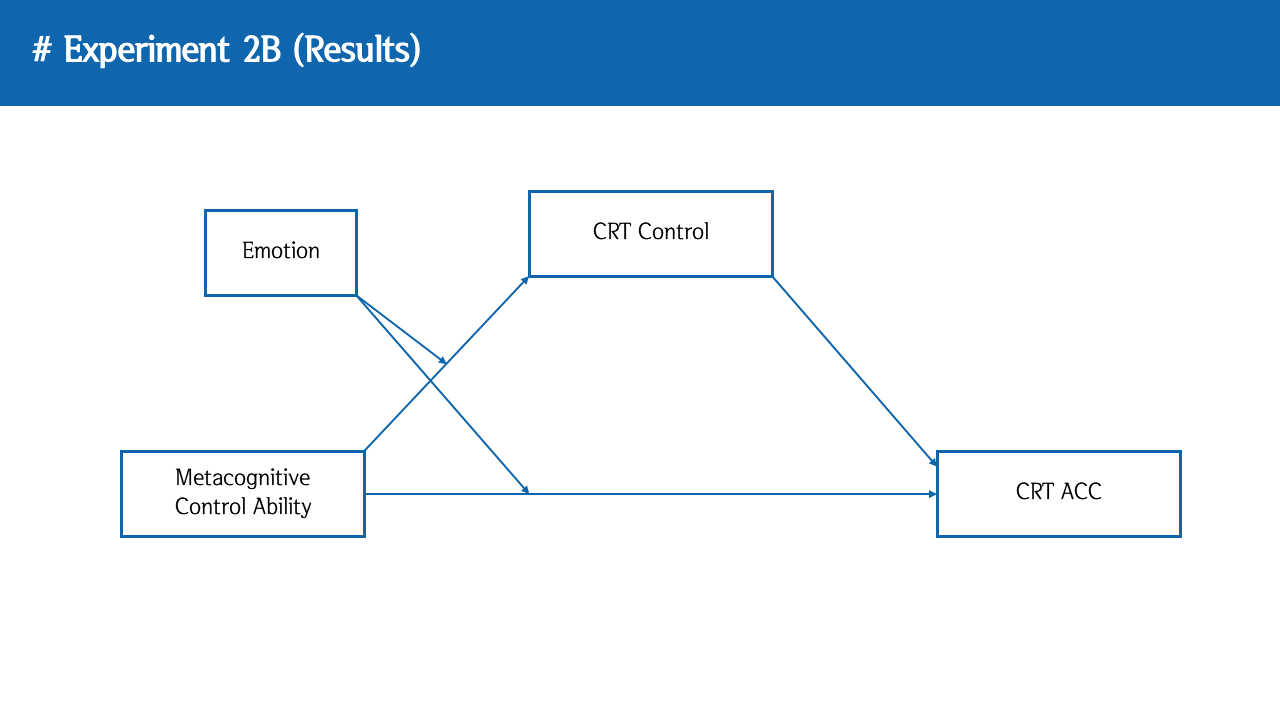


Supplementary Figure 8. Moderate mediation of emotion model 4 for control.


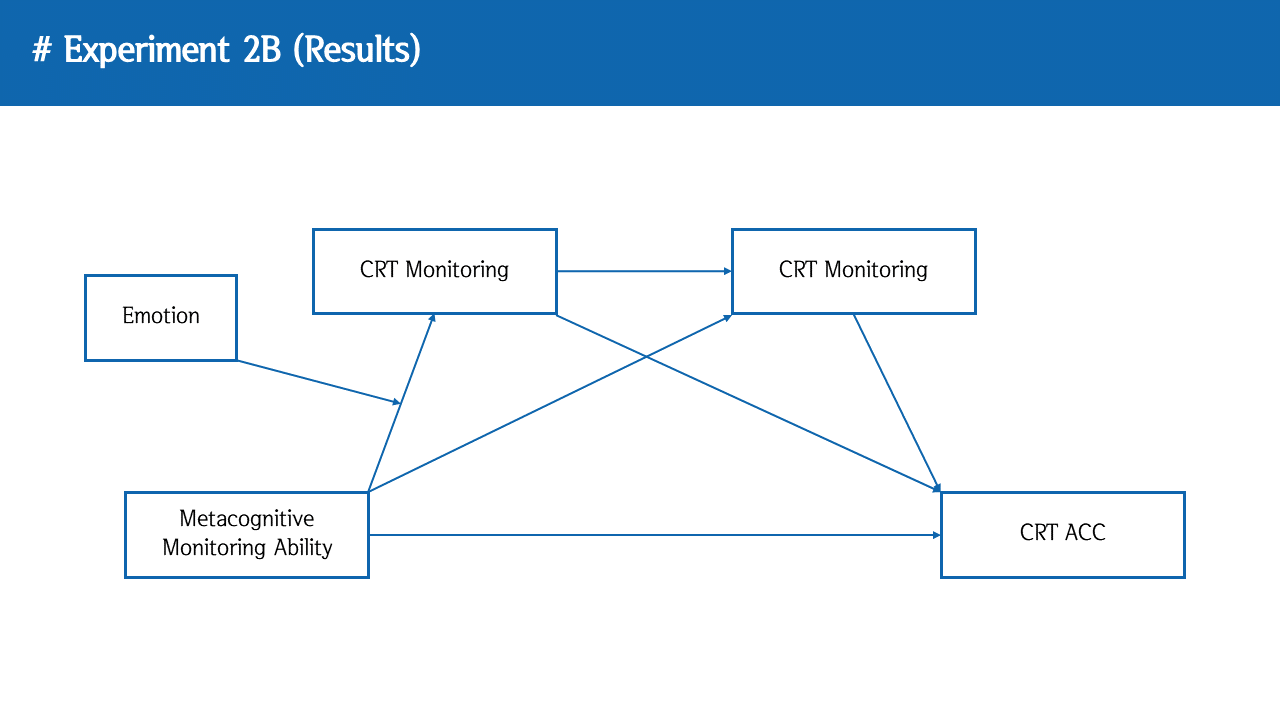


Supplementary Figure 9.


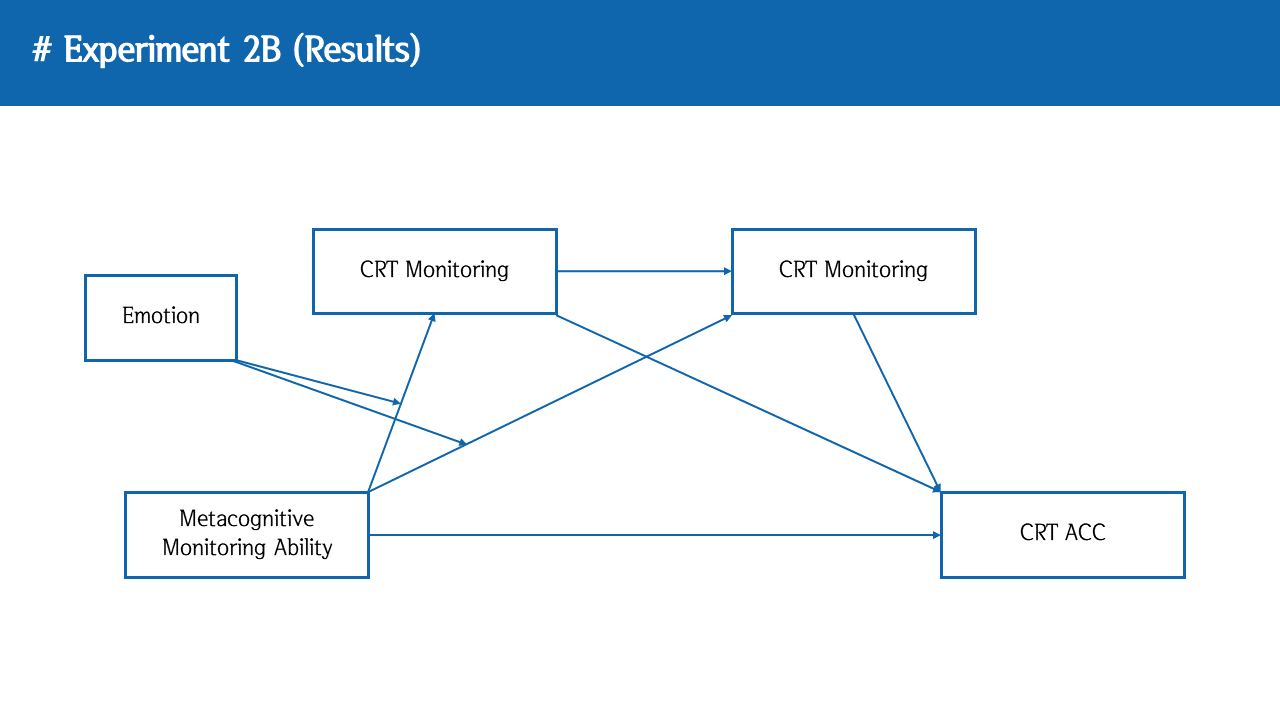


Supplementary Figure 10.

## Supplementary Tables

| **Dependent**  **variable** | **Low monitoring**  **(N=50)** | | | **High monitoring**  **(N=49)** | | | **t** |
| --- | --- | --- | --- | --- | --- | --- | --- |
|  | **M** | **SD** | **MSE** | **M** | **SD** | **MSE** |  |
| **Word-pair Task ACC** | .60 | .18 | .03 | .64 | .17 | .02 | 1.19 |
| **CRT ACC** | .43 | .22 | .03 | .52 | .19 | .03 | 2.20* |

** p<.05*

Supplementary Table 1. The results of word-pair task and CRT ACC in experiment 1.

|  | **Induced Emotions** | **Video contents** |
| --- | --- | --- |
| **Positive**  **emotions** | **Joyful** | A scene in which a husband who woke up from an anesthetic forgets being married and confesses his love to his wife. |
|  |  | A scene in which an excited baby and puppy watch as Dad is coming. |
|  | **Relieved** | A scene in which the sunshine forest and baby bear hug in mom's arms. |
|  |  | A scene in which peace is overcome in battle. |
| **Negative**  **emotions** | **Sad** | A scene in which a mother is sick and children have to live on their own. |
|  |  | A scene in which several friends torment a friend. |
|  | **Fearful** | A scene in which a murderer tricks a woman into the house. |
|  |  | A scene in which men are walking on a tightrope in a high sky. |

Supplementary Table 2. Explanation of each emotion video content.

|  | **Positive (N = 50)** | | **Negative (N = 54)** | | **t** |
| --- | --- | --- | --- | --- | --- |
|  | **M** | **SD** | **M** | **SD** |  |
| **Positive Emotional Level** | 5.53 | .84 | 1.51 | 1.16 | -20.35*** |
| **Negative Emotional Level** | 1.23 | .35 | 4.16 | 1.05 | 19.32*** |

** p<.05 **p<.01 ***p<.001*

Supplementary Table 3. The results of manipulation check in Experiment 2.

|  | **Low-monitoring** | | | **High-monitoring** | | | **Total** | | |
| --- | --- | --- | --- | --- | --- | --- | --- | --- | --- |
|  | **N** | **M** | **SD** | **N** | **M** | **SD** | **N** | **M** | **SD** |
| **Negative emotion** | 26 | .36 | .15 | 26 | .40 | .23 | 52 | .38 | .19 |
| **Positive emotion** | 25 | .38 | .13 | 25 | .12 | .12 | 50 | .25 | .18 |
| **Total** | 51 | .37 | .14 | 51 | .26 | .23 | 102 | .32 | .20 |

Supplementary Table 4. Descriptive statistics of CRT monitoring ability between the metacognitive monitoring ability groups and emotion manipulation in Experiment 2.

|  | **Low-control** | | | **High-control** | | | **Total** | | |
| --- | --- | --- | --- | --- | --- | --- | --- | --- | --- |
|  | **N** | **M** | **SD** | **N** | **M** | **SD** | **N** | **M** | **SD** |
| **Negative emotion** | 27 | .64 | .16 | 22 | .77 | .21 | 49 | .70 | .19 |
| **Positive emotion** | 23 | .60 | .23 | 27 | .32 | .22 | 50 | .45 | .26 |
| **Total** | 50 | .62 | .19 | 49 | .52 | .31 | 99 | .57 | .26 |

Supplementary Table 5. Descriptive statistics of CRT control ability between the metacognitive control ability groups and emotion manipulation in Experiment 2.

| **Variable** | **M (N=104)** | **SD** | **1** | **2** | **3** |
| --- | --- | --- | --- | --- | --- |
| 1. Metacognitive Monitoring | .09 | .05 | 1 |  |  |
| 1. CRT Monitoring | .31 | .20 | .11 | 1 |  |
| 1. CRT Accuracy | .36 | .23 | -.22* | -.43** | 1 |

Supplementary Table 6. Descriptive statistics and correlations among variables for model 1 & 2.

| **Predictor** | ***b*** | ***SE*** | ***t*** |
| --- | --- | --- | --- |
| **Partial effect of independent variables** | **CRT accuracy (CA)** | | |
| Metacognitive monitoring level (Meta M) | -.74 | .38 | -1.96 |
| CRT monitoring (CM) | -.42 | .11 | -3.97*** |
| Emotion (Emo) | -.07 | .04 | -1.54 |
| **Direct and total effects** |  |  |  |
| Meta M 🡺 CM | 1.89 | .52 | 3.62*** |
| Emo 🡺 CM | .35 | .07 | 5.09*** |
| Meta M * Emo 🡺 CM | -2.58 | .67 | -3.84*** |
| Meta M 🡺 CA | -.75 | .38 | -1.98* |
| CM 🡺 CA | -.47 | .10 | -4.64*** |
| ***R square (F value)*** | *.22 (13.89***)* | | |
| **Bootstrapping results for indirect effect** | **Estimate** | **SE** | **95% CI** |
| Meta M * Emo 🡺 CM 🡺 CA | -.26 | .21 | [-.79, .02] |

Note : CI = confidence interval. Bootstrap sample size = 5000.

* *p<*.05 ** *p<*.01 ****p<*.001

Supplementary Table 7. The results of regression and indirect effects for testing moderated mediation model 1.

| **Level** | | **Conditional indirect effect** | **Boot SE** | **95% CI** | |
| --- | --- | --- | --- | --- | --- |
|  |  |  |  | **LL** | **UL** |
| Emotion (Dummy) | Positive (0) | -.89 | .51 | -2.38 | -.41 |
|  | Negative (1) | .33 | .25 | -.08 | .94 |

Supplementary Table 8. Results of testing moderated mediation between conditional indirect effect for model 1.

| **Predictor** | ***b*** | ***SE*** | ***t*** |
| --- | --- | --- | --- |
| **Direct and total effects** |  |  |  |
| Meta M 🡺 CM | 1.89 | .52 | 3.62*** |
| Emo 🡺 CM | .35 | .07 | 5.09*** |
| Meta M * Emo 🡺 CM | -2.58 | .67 | -3.84*** |
| Meta M 🡺 CA | -.70 | .64 | -1.11 |
| Emo 🡺 CA | -.06 | .09 | -.67 |
| Meta M * Emo 🡺 CA | -.06 | .82 | -.07 |
| CM 🡺 CA | -.43 | .11 | -3.71*** |
| ***R square (F value)*** | *.23 (7.56***)* | | |
| **Bootstrapping results for indirect effect** | **Estimate** | **SE** | **95% CI** |
| Meta M * Emo 🡺 CM 🡺 CA | 1.10 | .56 | [.43, 2.64] |

Note : CI = confidence interval. Bootstrap sample size = 5000.

* *p<*.05 ** *p<*.01 ****p<*.001

Supplementary Table 9. The results of regression and indirect effects for testing moderated mediation model 2.

| **Level** | | **Conditional indirect effect** | **Boot SE** | **95% CI** | |
| --- | --- | --- | --- | --- | --- |
|  |  |  |  | **LL** | **UL** |
| Emotion (Dummy) | Positive (0) | -.80 | .47 | -2.19 | -.35 |
|  | Negative (1) | .29 | .22 | -.07 | .82 |

Supplementary Table 10. Results of testing moderated mediation between conditional indirect effect for model 2.

| **Variable** | **M (N=104)** | **SD** | **1** | **2** | **3** |
| --- | --- | --- | --- | --- | --- |
| 1. Metacognitive Control | .09 | .05 | 1 |  |  |
| 1. CRT Control | .31 | .20 | .11 | 1 |  |
| 1. CRT Accuracy | .36 | .23 | -.22* | -.43** | 1 |

Supplementary Table 11. Descriptive statistics and correlations among variables for model 3 &4.

| **Predictor** | ***b*** | ***SE*** | ***t*** |
| --- | --- | --- | --- |
| **Partial effect of independent variables** | **CRT accuracy (CA)** | | |
| Metacognitive control level (Meta C) | .01 | .11 | .06 |
| CRT control (CC) | -.35 | .09 | -3.79*** |
| Emotion (Emo) | -.04 | .05 | -.75 |
| **Direct and total effects** |  |  |  |
| Meta C 🡺 CC | .67 | .16 | 4.33*** |
| Emo 🡺 CC | .56 | .09 | 6.19*** |
| Meta C * Emo 🡺 CC | -.86 | .22 | -3.93*** |
| Meta C 🡺 CA | .01 | .11 | .08 |
| CC 🡺 CA | -.38 | .08 | -4.73*** |
| ***R square (F value)*** | *.19 (11.59***)* | | |
| **Bootstrapping results for indirect effect** | **Estimate** | **SE** | **95% CI** |
| Meta C * Emo 🡺 CC 🡺 CA | .33 | .11 | [.15, .58] |

Note : CI = confidence interval. Bootstrap sample size = 5000.

* *p<*.05 ** *p<*.01 ****p<*.001

Supplementary Table 12. The results of regression and indirect effects for testing moderated mediation model 3.

| **Level** | | **Conditional indirect effect** | **Boot SE** | **95% CI** | |
| --- | --- | --- | --- | --- | --- |
|  |  |  |  | **LL** | **UL** |
| Emotion (Dummy) | Positive (0) | -.26 | .09 | -.46 | -.12 |
|  | Negative (1) | .07 | .06 | -.03 | .19 |

Supplementary Table 13. Results of testing moderated mediation between conditional indirect effect for model 3.

| **Predictor** | ***b*** | ***SE*** | ***t*** |
| --- | --- | --- | --- |
| **Direct and total effects** |  |  |  |
| Meta C 🡺 CC | .67 | .16 | 4.33*** |
| Emo 🡺 CC | .56 | .09 | 6.19*** |
| Meta C * Emo 🡺 CC | -.86 | .22 | -3.93*** |
| Meta C 🡺 CA | .01 | .11 | .08 |
| Emo 🡺 CA | .14 | .10 | 1.33 |
| Meta C * Emo 🡺 CA | -.43 | .23 | -1.87 |
| CC 🡺 CA | -.41 | .10 | -4.25*** |
| ***R square (F value)*** | *.22 (6.93***)* | | |
| **Bootstrapping results for indirect effect** | **Estimate** | **SE** | **95% CI** |
| Meta C * Emo 🡺 CC 🡺 CA | .36 | .13 | [.15, .65] |

Note : CI = confidence interval. Bootstrap sample size = 5000.

* *p<*.05 ** *p<*.01 ****p<*.001

Supplementary Table 14. The results of regression and indirect effects for testing moderated mediation model 4.

| **Level** | | **Conditional indirect effect** | **Boot SE** | **95% CI** | |
| --- | --- | --- | --- | --- | --- |
|  |  |  |  | **LL** | **UL** |
| Emotion (Dummy) | Positive (0) | -.28 | .10 | -.50 | -.12 |
|  | Negative (1) | .08 | .06 | -.02 | .22 |

Supplementary Table 15. Results of testing moderated mediation between conditional indirect effect for model 4.

| **Variable** | **M (N=104)** | **SD** | **1** | **2** | **3** | **4** |
| --- | --- | --- | --- | --- | --- | --- |
| 1. Metacognitive Monitoring | .09 | .05 | 1 |  |  |  |
| 1. CRT Monitoring | .31 | .20 | .11 | 1 |  |  |
| 1. CRT control | .58 | .26 | .20* | .47*** | 1 |  |
| 1. CRT accuracy | .36 | .23 | -.22* | -.43** | -.43** | 1 |

** p<.05 **p<.01 ***p<.001*

**Supplementary Table 16. Descriptive statistics and correlations among variables in model 5 & 6.**

| **Predictor** | ***b*** | ***SE*** | ***t*** |
| --- | --- | --- | --- |
| **Partial effect of independent variables** | **CRT accuracy (CA)** | | |
| Metacognitive monitoring level (Meta M) | -.59 | .37 | -1.58 |
| CRT monitoring (CM) | -.33 | .11 | -2.89** |
| CRT control (CC) | -.22 | .09 | -2.29* |
| Emotion (Emo) | -.02 | .05 | -.53 |
| **Direct and total effects (Model 5)** |  |  |  |
| Meta M 🡺 CM | 1.89 | .52 | 3.62*** |
| Emo 🡺 CM | .35 | .07 | 5.09*** |
| Meta M * Emo 🡺 CM | -2.58 | .67 | -3.84*** |
| Meta M 🡺 CC | .72 | .42 | 1.70 |
| CM 🡺 CC | .60 | .11 | 5.24*** |
| Meta M 🡺 CA | -.58 | .37 | -1.56 |
| CM 🡺 CA | -.33 | .11 | -2.98** |
| CC 🡺 CA | -.24 | .09 | -2.74** |
| **Model 1** $R^{2}$ **(*F*)** | .27 (12.35***) | | |
| **Bootstrapping results for indirect effect** | **Estimate** | **SE** | **95% CI** |
| Meta M * Emo 🡺 CM 🡺 CC 🡺 CA | .36 | .24 | [.07, 1.00] |

Note : CI = confidence interval. Bootstrap sample size = 5000.

* *p<*.05 ** *p<*.01 ****p<*.001

Supplementary Table 17. Regression and indirect effects results for model 5.

| **Predictor** | ***b*** | ***SE*** | ***t*** |
| --- | --- | --- | --- |
| **Direct and total effects (Model 6)** |  |  |  |
| Meta M 🡺 CM | 1.89 | .52 | 3.62*** |
| Emo 🡺 CM | .35 | .07 | 5.09*** |
| Meta M * Emo 🡺 CM | -2.58 | .67 | -3.84*** |
| Meta M 🡺 CC | 2.41 | .62 | 3.86*** |
| CM 🡺 CC | .31 | .11 | 2.75** |
| Emo 🡺 CC | .45 | .09 | 5.21*** |
| Meta M * Emo 🡺 CC | -2.78 | .81 | -3.44*** |
| Meta M 🡺 CA | -.58 | .37 | -1.56 |
| CM 🡺 CA | -.33 | .11 | -2.98** |
| CC 🡺 CA | -.24 | .09 | -2.74** |
| **Model 1** $R^{2}$ **(*F*)** | .27 (12.35***) | | |
| **Bootstrapping results for indirect effect** | **Estimate** | **SE** | **95% CI** |
| Meta M * Emo 🡺 CM 🡺 CC 🡺 CA | .19 | .14 | [.00, .51] |

Note : CI = confidence interval. Bootstrap sample size = 5000.

* *p<*.05 ** *p<*.01 ****p<*.001

Supplementary Table 18. Regression and indirect effects results for model 6.

| **Model** | **Level** | | **Conditional indirect effect** | **Boot SE** | **95% CI** | |
| --- | --- | --- | --- | --- | --- | --- |
|  |  |  |  |  | **LL** | **UL** |
| **5** | Emotion  (Dummy) | Positive (0) | -.27 | .19 | -.81 | -.06 |
|  |  | Negative (1) | .10 | .09 | -.02 | .32 |
| **6** | Emotion  (Dummy) | Positive (0) | -.14 | .10 | -.39 | -.00 |
|  |  | Negative (1) | .05 | .05 | -.01 | .19 |

Supplementary Table 19. Results of testing moderated mediation between conditional indirect effect model 5 & 6.

|  | **M (sec)** | **SD** | **t (*p*)** |
| --- | --- | --- | --- |
| **Correct** | 24.91 | 22.31 | -.27 (*.79*) |
| **Incorrect** | 24.41 | 23.27 |  |

Supplementary Table 20. Analysis for taking time between correct / incorrect questions.
